# Therapeutic Efficacy of Difluprednate 0.05% Versus Prednisolone Acetate 1% in Controlling Inflammation and Macular Oedema Following Phacoemulsification: An Optical Coherence Tomography-Based Study

**DOI:** 10.7759/cureus.14673

**Published:** 2021-04-25

**Authors:** Bijnya B Panda, Ashok Nanda, Suresh C Swain

**Affiliations:** 1 Ophthalmology, Srirama Chandra Bhanja Medical College and Hospital, Cuttack, IND; 2 Ophthalmology, Kar Vision Eye Hospital, Bhubaneswar, IND

**Keywords:** phacoemulsification, postoperative inflammation, difluprednate, macular edema

## Abstract

Background: Topical corticosteroids have been the cornerstone in the management of postoperative inflammation following cataract surgery. Due to potential side effects of the older topical steroids like prednisolone acetate and dexamethasone or betamethasone, newer potent steroids preparation like difluprednate, loteprednol or fluorometholone are now being used at lesser dose and frequency to control inflammation. There is scanty literature on the efficacy of these drugs in preventing inflammation and macular oedema in the Indian population.

Purpose: The purpose of this study was to compare the efficacy of difluprednate 0.05% against prednisolone 1% eye drops for control of inflammation following phacoemulsification. The adverse effects of both drugs were also evaluated in this retrospective study.

Methods: This retrospective cohort study included 181 patients operated for age-related cataracts by a single surgeon at a tertiary referral eye hospital between December 2018 and March 2019. Patients received either difluprednate 0.05% emulsion (n=90 eyes) or prednisolone acetate 1% (n=91 eyes) after phacoemulsification with the same brand intraocular lens (IOL) and same phaco machine. The topical medication was initiated one day postoperatively and continued for six weeks in tapering dosage. Pain score (Visual Analogue Scale, VAS), conjunctival hyperemia, anterior chamber (AC) cell grading, corneal oedema, central retinal thickness, subclinical cystoid macular oedema (SCME), intraocular pressures (IOP) and best-corrected visual acuity (BCVA) were examined after one week and six weeks of cataract extraction.

Results: There was no statistically significant difference observed with regards to pain score (no pain in any patients after six weeks), conjunctival hyperemia (no hyperemia in any patients after six weeks), AC inflammation (no reaction in any patients), central retinal thickness (234.44 ± 35.75µ vs. 234.8 ± 34.99µ, p-value 0.946), SCME (16.67% vs. 13.19%, p-value 0.511), IOP (16.8 vs. 15.47 mmHg, p-value 0.101) and BCVA (BCVA 6/6 in 57.7% vs. 70.32%, p-value >0.05) between both groups. The mean change in IOP in both the groups at one week (0 ± 4.4 vs. 1.87 ± 3.54, p-value 0.0007) and six weeks (−0.01 ± 5.53 vs. 1.88 ± 4.01, p-value 0.004) was significant.

Conclusion: Both the groups were equivalent with regards to their therapeutic efficacy in controlling postoperative inflammation and restoration of vision following phacoemulsification.

## Introduction

The latest, safest and most popular cataract extraction technique performed worldwide is phacoemulsification with posterior chamber intraocular lens implantation [1]. It involves minimal ultrasonic power and surgical tissue manipulation, lesser postoperative inflammation and early visual restoration [1]. However, the mechanical and ultrasonic energy induces breakdown of the ocular blood-aqueous barrier and thereby can potentially cause inflammation which manifests as conjunctival hyperemia, anterior chamber (AC) reaction, raised intraocular pressure (IOP) and cystoid macular oedema (CME) [2]. Topical corticosteroids remain the gold-standard drug to treat such inflammation following cataract surgery [3-7]. Due to the potential adverse effects of the older topical steroids (prednisolone acetate, dexamethasone and betamethasone) on IOP and posterior sub-capsular cataract formation, newer potent steroid preparations like difluprednate, loteprednol and fluorometholone are preferred [8]. There are few studies to date regarding the efficacy of difluprednate 0.05% in the treatment of endogenous uveitis [9,10] and postsurgical inflammation [11-14].

Difluprednate is a prednisolone derivative with structural modifications (fluorination at C6 and C9 positions and replace the 17-hydroxyl group with butyric acid). It has better anti-inflammatory activity compared to prednisolone acetate, dexamethasone and betamethasone [15]. The Food and Drug Administration (FDA) approved difluprednate 0.05% ophthalmic suspension in 2008 for use in postoperative inflammation and pain of adults [16]. However, few studies have demonstrated its efficacy even in young children (zero to three years) to manage postoperative inflammation following cataract surgery [17]. Difluprednate exhibits better tissue penetration, bioavailability, rapid local metabolism and strong efficacy with less incidence of rising IOP [18]. It can be used in less frequent dosage (two to four times daily), leading to better compliance.

Optical coherence tomography (OCT) recording after phacoemulsification has shown a significant increase in the retinal thickness of the fovea in the postoperative period and returns to the previous levels by 28 days [19]. However, if this persists for a long time, it causes intraretinal cystic spaces, otherwise called subclinical cystoid macular oedema (SCME) [19,20]. The postsurgical inflammation in the macula following topical steroid administration has not been studied adequately using OCT [19-21]. Few studies on spectral-domain OCT documented the resolution of pseudophakic CME after administration of topical difluprednate [19-21]. To the best of our knowledge, there is only one comparative study between difluprednate 0.05% and prednisolone acetate in the postphacoemulsification period using OCT [21]. Palacio-Pastrana et al. did not find any difference in central retinal thickness, conjunctival hyperemia, oedema, pain and photophobia between the two drugs [21]. Accordingly, the present study was designed to compare the efficacy and safety of difluprednate 0.05% emulsion versus 1% prednisolone acetate in controlling SCME using swept-source OCT.

## Materials and methods

The present retrospective cohort study was conducted in a tertiary referral eye hospital. All patients operated for age-related cataracts with phacoemulsification under topical anaesthesia by a single surgeon between December 2018 and March 2019 were included. A total of 181 eyes fulfilling the inclusion criteria (phacoemulsification with clear corneal incision and implanted foldable posterior chamber intraocular lens, completing a follow-up of six weeks) were taken into consideration. Eyes with a history of chronic ocular inflammation, glaucoma, corneal disease, previous ocular surgery, any retinal pathologies, or intra-operative complications (posterior capsular rent, nucleus drop, vitreous loss, etc.) were excluded from the study.

Patients who received difluprednate 0.05% and prednisolone 1% were categorised as group A (n=90) and group B (n=91) respectively. Baseline preoperative evaluation for best-corrected visual acuity (BCVA), IOP, central retinal thickness (CRT), dilated fundoscopy, lacrimal stringing (to rule out chronic dacryocystitis), viral markers (HIV, HbsAg and HCV), blood pressure, and random blood sugar was done. Phacoemulsification (clear corneal incision) with foldable posterior chamber IOL implantation was carried out in all patients by a single experienced surgeon. The primary outcome measures were AC cells grading and CRT after one week and six weeks postoperatively. CRT was measured with the help of Swept-source OCT [DRI Triton Topcon, SS-OCT (Hasunuma-Cho, Itabashi-Ku, Tokyo, Japan)] using a 6 mm cube scan centred on the fovea. Secondary outcome measures were ocular pain, drug tolerability, conjunctival hyperemia, corneal oedema, IOP, and BCVA at one week and six weeks postoperatively.

Dosage of administration

Difluprednate 0.05% eye drop 3 times/day × 2 weeks, 2 times/day × 2 weeks and once/day × 2 weeks. Prednisolone 1% eye drops 6 times a day, on weekly tapering doses for 6 weeks.

The primary outcome measures (AC cells grading and CRT) and secondary outcome measures (ocular pain score, drug tolerance grading, slit-lamp findings) were assessed in Table 1.

**Table 1:** Assessment of primary and secondary outcome measures. (A) Ocular pain assessment using a VAS, (B) drug tolerance grading based on our own criteria of patient discomfort and (C) evaluation of conjunctival hyperaemia, corneal oedema and anterior chamber with slit-lamp. SUN: Standardization of Uveitis nomenclature [25], DM: Descemet's membrane, VAS: visual analogue scale, AC: anterior chamber, HPF: high power field.

(A) Ocular pain score (VAS) [22]		
0	No pain	
1-3	Mild pain	
4-6	Moderate-severe pain	
7-9	Very severe pain	
10	Worst pain	
(B) Drug tolerance grading (eye symptoms)		
0	No discomfort	
1	Mild stinging for a few seconds	
2	Stinging for 30-60 seconds	
3	Stinging persisted ≥60 seconds	
(C) Slit-lamp findings		
Conjunctival hyperaemia (as per Japan Ocular Allergy Society grade standard photograph [23])		
0	No vasodilation	
1	Some vasodilation	
2	Extensive vasodilation	
3	Overall vasodilation	
Corneal oedema (graded from 0 to 3) [24]		
0	Clear cornea	
1	Mild oedema, evident by DM fold	
2	Moderate oedema, evident as DM fold with stromal oedema of a part of cornea	
3	Severe oedema causing difficulty in the view of the anterior chamber and iris pattern	
Aqueous cells and flare in AC according to SUN classification scheme (grades 0 to 4) [25]		
Grade	Cells/HPF	Flare
0	<1	None
0.5+	1-5	None
1+	6-15	Faint
2+	16-25	Moderate (iris/lens details clear)
3+	26-50	Marked (iris/lens hazy)
4+	>50	Intense (fibrin or plastic aqueous)

The CRT was measured by Swept-source OCT [DRI Triton Topcon, SS-OCT (Hasunuma-Cho, Itabashi-Ku, Tokyo, Japan)]. An increase in CRT or presence of cystic spaces on OCT without a decrease in BCVA was considered to be significant and defined as subclinical CME [19,26]. The BCVA was recorded in Snellen’s chart and converted to Log MAR for analysis. The IOP was measured with the help of a noncontact Pneumotonometer (all patients had an average corneal thickness, 520-545 µm).

Statistical analysis

Data entered were analyzed using SPSS software (version 21.0, IBM Corp., Armonk, NY). Quantitative variables were compared between the two groups using the Mann-Whitney test, and Wilcoxon signed-rank test was used for comparison between pre- and postoperative variables. Qualitative variables were compared using the Chi-square test/Fisher’s exact test. The p-value of <0.05 was considered statistically significant. Yates correction was applied wherever the frequencies of sub-categories were less than 5.

## Results

A total of 181 patients were included (difluprednate 0.05%, n=90; prednisolone acetate 1%, n=91) in this study; of which 58 patients were <60 years, 110 in the age group 61-80 years, and 13 patients were >80 years. In group A, out of 90 patients, 57 (63.33%) were males and 33 (36.67%) were females. In group B, there were 60 males (65.93%) and 31 females (34.07%). The mean age of groups A and B was 63.29 ± 11.3 and 65.36 ± 8.97, respectively (t-test, p=0.173).

Primary outcome measures

Anterior Chamber Cells and Flare

The AC reaction at one week postoperatively in group A showed an equal number of patients having grade 0 (n=44, 48.9%) and grade 1 (n=44, 48.9%) aqueous cells. There were 2.2% (n=2) of patients with grade 2 aqueous cells. In group B, the majority of patients (n=48, 52.7%) showed grade 0 while 46.2% (n=42) had grade 1 and 1.1% (n=1) had grade 2 aqueous cells. The intragroup comparison showed that the difference in controlling AC reaction was not statistically significant (p=0.292, Chi-square test). At the end of six weeks, none of the patients in both groups showed any reaction.

CRT

The mean postoperative CRT measured at one week was 225.88 ± 34.84 µm and 224.57 ± 31.22 µm (t-test, p=0.79) and at six weeks were 234.44 ± 35.75 µm and 234.8 ± 34.99 µm (t-test, p=0.946) in groups A and B, respectively. There was no significant difference in CRT between the two groups indicating equivalent efficacy of topical difluprednate and prednisolone acetate in reducing subclinical CME. Subclinical macular oedema at one week postoperatively was seen in 9/90 patients (10%) in group A as compared to 5/91 (5.49%) in group B (p=0.257, Chi-square test). Similarly, at the end of six weeks, macular oedema was seen in 15 patients (16.67%) in group A and 12 patients (13.19%) in group B (p=0.511, Chi-square test). Comparing the percentage of occurrence of macular oedema between the two groups by Chi-square test, the results were statistically not significant.

Secondary outcome measures

Ocular Pain Score and Drug Tolerance

Most of the patients (n=169, 93.37%) had no pain after one week except for grade 1 pain in eight patients (8.89%) of group A and four patients (4.4%) of group B (p=0.249, Fisher Exact test). After six weeks, none of the patients complained of any ocular pain in both groups.

At the end of one week, there was no ocular discomfort in 93.33% (n=84) and 98.9% (n=90) of patients in groups A and B, respectively (p=0.066, Chi-square test). Four patients had mild stinging for <30 seconds (grade 1) and two patients had stinging for 30-60 seconds (grade 2) in group A. One patient (grade 3) in group B needed change in the drug because of severe discomfort in the eye. None of the patients experienced any form of drug discomfort at the end of six weeks.

Conjunctival Hyperemia

The majority of patients (n=59, 65.66%) in group A did not show conjunctival hyperemia; 30 patients (33.33%) showed grade 1 conjunctival hyperemia and only one patient showed grade 2 hyperemia. A relatively higher percentage of patients (n=69, 75.82%) were congestion-free in group B after one week. However, statistically, this difference was not significant (p=0.222, Chi-square test). None of the patients showed conjunctival hyperemia at the end of six weeks.

Corneal Oedema

The corneal clarity at the end of the first week in both the groups was similar (p=0.194, Chi-square test). Only two patients in group A showed grade 2 oedema while none of the patients in group B had corneal oedema. At the end of six weeks, only one patient in group A showed persistent corneal oedema, the cause could be attributed to rising IOP in the follow-up period.

IOP

The mean IOP rise at one week and six weeks after administration of difluprednate were 0 ± 4.4 mmHg and 0.01 ± 5.53 mmHg, respectively, whereas, in group B, it was 1.87 ± 3.54 mmHg and 1.88 ± 4.01 mmHg, respectively, suggesting a higher rise in IOP in group B. The change in IOP in both the groups after one week and six weeks were statistically significant (p=0.0007 and 0.004, Mann Whitney test). However, there was no significant difference in IOP between the groups in the preoperative period and postoperative periods. There were 16/90 (17.7%) patients in the difluprednate-treated group and 9/91 (9.8%) patients in the prednisolone-treated group, who had IOP of > 21 mmHg (Tables 2 and 3).

**Table 2:** Comparison of intraocular pressure (mmHg) between difluprednate and prednisolone groups.

IOP (mmHg)	Group A Diflucor (n=90)	Group B Predforte (n=91)	P-value
Preoperative			
Mean ± Stdev	16.79 ± 3.41	17.35 ± 2.93	0.174 (Mann Whitney test)
Range	10-26	10-25	
Postoperative one week			
Mean ± Stdev	16.79 ± 4.66	15.48 ± 3.63	0.063 (Mann Whitney test)
Range	9-34	8-31	
Postoperative six weeks			
Mean ± Stdev	16.8 ± 5.17	15.47 ± 4.43	0.101 (Mann Whitney test)
Range	8-29	8-30	

**Table 3:** Comparison of change in intraocular pressure (mmHg) between difluprednate and prednisolone groups.

Change in IOP (mmHg)	Group A Diflucor (n=90)	Group B Predforte (n=91)	P-value
Postoperative one week			
Mean ± Stdev	0 ± 4.4	1.87 ± 3.54	0.0007 (Mann Whitney test)
Range	−16 to 12	−11 to 8	
Postoperative six weeks			
Mean ± Stdev	−0.01 ± 5.53	1.88 ± 4.01	0.004 (Mann Whitney test)
Range	−15 to 12	−15 to 10	

BCVA

There were 52 patients (57.7%) with a final BCVA of 6/6 and 31 patients (34.4 %) with BCVA 6/9 in group A, whereas 64 patients (70.32%) had BCVA 6/6 and 20 patients (21.9%) had BCVA 6/9 in group B. There was no statistically significant difference among patients with BCVA>6/9 in both the groups at the end of the study with X^2^=0.0659 (with Yates correction) and P=0.797 (p-value<0.05).

## Discussion

Newer techniques in phacoemulsification and the advent of safer topical steroids and non-steroidal drugs (NSAIDs) administration have led to quick visual rehabilitation after cataract surgery. Yet, postoperative inflammation in the form of AC reaction and CME continues to be the major factors causing sub-optimal visual outcomes [27]. Corticosteroids play a vital role in the treatment of this inflammation by inhibiting the release of arachidonic acid from cell membrane phospholipids and prevent the production of leukotrienes and prostaglandins. Older potent topical corticosteroids like prednisolone acetate and dexamethasone are now being replaced with newer and safer steroids like difluprednate and fluorometholone [27]. The known advantages of difluprednate related to its better patient compliance over prednisolone are its better bioavailability, enhanced penetration and dose uniformity apart from being preservative-free. But the efficacy of difluprednate in reducing pseudophakic SCME using OCT has never been studied before in the Indian population. Therefore, this study aimed to compare the efficacy of topical difluprednate 0.05% versus topical prednisolone acetate 1% in controlling postoperative inflammation and SCME after uncomplicated phacoemulsification as demonstrated by Swept-source OCT. Recently, the Swept-source OCT has been found to have revolutionized the management of CME due to its enhanced speed, accuracy, and higher resolution characteristics.

In the present study, we found that the efficacy in controlling AC inflammation after cataract surgery in both difluprednate and prednisolone groups was equivalent. This is in accordance with the study by Saman et al. who showed that difluprednate is not inferior to prednisolone in reducing AC cells and flare but the resolution of signs of ocular inflammation was faster in the prednisolone group after one week of treatment [15]. 

No significant differences in the analysis of AC cells and flare were observed between the two drug groups at any time of follow-up in a multicenter RCT studied in Mexico [21]. Khalaf Allah et al., in their systematic review, have reported that after small incision cataract surgery, difluprednate was superior in clearing AC cells at one week (OR=2.5, p>0.00001) and at two weeks (OR=2.5, p=0.04), as well as clearing the AC flare at two weeks (OR=6.7, p=0.04). They also found difluprednate to be superior in terms of corneal clarity at one day (OR=2.6, p=0.02) and one week after phacoemulsification surgery (OR=1.96, p=0.0007). However, both drugs were equally effective at one month [27].

Our study revealed that prednisolone could better reduce the conjunctival hyperemia in the first week of surgery, but the difference was not statistically significant. The pain score and drug discomfort were similar in both groups during the postoperative period. There was no significant difference in the reduction of corneal oedema among both groups. Similar to the study of Gurg et al., we did not observe any difference in BCVA between the groups at the end of six weeks. However, only 57.7% of patients receiving difluprednate had BCVA of 6/6 in our study compared to 90% of patients in the study of Garg et al. [13]. These differences in final BCVA may be due to variation in patient-related characteristics and surgical techniques.

In the present study, the mean IOP (six weeks postop) in difluprednate treated patients was 16.8 ± 5.17 mm Hg and 15.47 ± 4.43 in the prednisolone-treated patients. There were about 18% patients with IOP of more than 21 mmHg in the difluprednate group compared to 10% of the prednisolone group. This finding was similar to Kusne et al. who reported that patients who received difluprednate showed a statistically significant increase in IOP postoperatively as compared to those on prednisolone [28]. Our study results do not match with Garg et al. [13] who have reported that none of their patients had IOP >21 mmHg, whereas the Mexico trial has shown a transient rise in IOP in two patients in the difluprednate treated group that returned to baseline at six weeks [21].

There are certain limitations in this study. The retrospective study design and small sample size are major limitations. The data on phacoemulsification time and power were not collected. These parameters might affect postoperative inflammation, corneal oedema and macula thickness. We have used the SUN classification for Slit-lamp grading of AC cells and flare instead of using Laser Flare cell photometry (LFCP) due to the unavailability of equipment which has been recommended as the most sensitive and reproducible method as suggested by De Maria et al. [25,29]. However, the SUN classification is worldwide accepted and validated.

## Conclusions

Both the drugs are equivalent in their efficacy in reducing postoperative inflammation following phacoemulsification. The difluprednate group may cause a higher rise in IOP, and therefore should be used judiciously in patients with previous ocular hypertension and steroid responders. Difluprednate has been successfully added to the group of anti-inflammatory drugs in postcataract surgery for the past few years and remains to be so. The added advantages of being BAK-free and less frequent dosing make it more compliant and tolerable in patients.
